# Maternal serum iron status, hepcidin and interleukin-6 levels in women with preeclampsia

**DOI:** 10.3389/fphys.2023.1049994

**Published:** 2023-02-24

**Authors:** Yasir I. B. Ahmed, Hind S. Yagoub, Mohamed A. Hassan, I. Adam, Hamdan Z. Hamdan

**Affiliations:** ^1^ Faculty of Medical Laboratory Sciences, Omdurman Islamic University, Omdurman, Sudan; ^2^ Faculty of Medical Laboratory Sciences, College of Applied Medical Sciences, Taibah University, Madinah, Saudi Arabia; ^3^ Africa City of Technology, Khartoum, Sudan; ^4^ Department of Obstetrics and Gynaecology, Unaizah College of Medicine and Medical Sciences, Qassim University, Unaizah, Saudi Arabia; ^5^ Department of Basic Medical Sciences, Unaizah College of Medicine and Medical Sciences, Qassim University, Unaizah, Saudi Arabia; ^6^ Faculty of Medicine, Al-Neelain University, Khartoum, Sudan

**Keywords:** preeclampsia, hepcidin, iron homeostasis, interleukin-6, sub-Sahara Africa, pregnancy

## Abstract

**Introduction:** Preeclampsia can lead to a number of adverse maternal and perinatal effects. The association between iron status [serum iron, ferritin and total iron-binding capacity (TIBC)], unsaturated iron-binding capacity, hepcidin, interleukin-6 (IL-6) levels and preeclampsia is not fully understood.

**Objective:** To assess the levels of iron status, hepcidin and interleukin-6 in women with preeclampsia compared with healthy pregnant women.

**Method:** A case-control study (60 women were recruited in each group) was conducted at Saad Abuelela Maternity Hospital in Khartoum, Sudan. Sociodemographic and clinical data were gathered through a questionnaire. The levels of iron status, hepcidin and IL-6 were measured using applicable methods.

**Results:** There was no significant difference in the median [interquartile range (IQR)] of age, parity or body mass index between the two groups. Moreover, the median (IQR) of the iron status, hepcidin and interleukin-6 did not differ between women with preeclampsia and healthy controls. There were no significant correlations between haemoglobin, hepcidin and IL-6. There were also no significant correlations between serum iron, serum ferritin, hepcidin and IL-6. However, there was a significant positive correlation between hepcidin and IL-6 (r = 0.393, *p* = 0.002).

**Conclusion:** In this study, women with preeclampsia had levels of iron status, hepcidin and IL-6 similar to those observed in healthy pregnant women. There was no significant correlation between iron status, hepcidin and IL-6.

## Introduction

Preeclampsia is a multiorgan disease which occurs in the second half of pregnancy (i.e., after the 20th week of gestation), and it is common in pregnancy-related disorders (ACOG Committee on Obstetric Practice, 2002). It is a global health problem that affects 2%–8% of pregnant/parturient women (Abalos et al., 2013). Preeclampsia is the leading cause of maternal and perinatal adverse effects (Abalos et al., 2013). Several obstetrics, clinical, biochemical, nutritional and immunological factors have been identified as risk factors for preeclampsia (Meazaw et al., 2020).

Although the exact pathophysiology of preeclampsia remains to be understood, preeclampsia is characterised by poor placentation (shallow cytotrophoblast invasion), which results in defective remodelling of the maternal spiral arteries (Hoodbhoy and Payne, 2016). Poor placentation (under-perfused placenta) can lead to maternal angiogenic imbalance and results in activation of the inflammatory response (Hoodbhoy and Payne, 2016).

Hepcidin is a peptide hormone that plays an essential role in iron status to keep it tightly regulated for haemoglobin and erythropoiesis without allowing iron overload to occur in the body. It is an acute-phase reactant that can increase in response to inflammation (Chambers et al., 2021). It is expected that hepcidin is lower during pregnancy to enhance optimum iron bioavailability to the mother as well as for fetus. However, inflammatory states during pregnancy such as preeclampsia are reported to be associated with higher hepcidin levels during pregnancy (Koenig et al., 2014). The proposed elevated hepcidin level in women with preeclampsia could act as a protective mechanism to counteract iron overload (mediated cytotoxicity), oxidative stress and endothelial dysfunction that might occur in women with preeclampsia (Shaji Geetha et al., 2022).

The association between the levels of iron status, hepcidin and interleukin-6 (IL-6), and preeclampsia is not fully understood. Some studies have reported higher levels of iron status, hepcidin and IL-6 (Muhsin et al., 2016; Brunacci et al., 2018; Shaji Geetha et al., 2022; Ölmez et al., 2022) in healthy controls, while other studies have shown no significant difference in these elements in women with preeclampsia and controls (Duvan et al., 2015; Cardaropoli et al., 2018). Moreover, none of these studies were conducted in sub-Saharan Africa, including Sudan. In sub-Saharan Africa, there is a high prevalence of anaemia among pregnant women (Geta et al., 2022), which might have an effect on the association between iron status, hepcidin, IL-6 and preeclampsia. Likewise, the pathophysiology of preeclampsia might be different in Africa from other settings for a number of reasons, for example, communicable diseases such as malaria (Adam et al., 2011) and bacterial (Ahmed et al., 2020) and viral infections (Ahmed et al., 2018), which have been reported to be associated with preeclampsia in sub-Saharan Africa. Therefore, there is a need to assess the association between iron status, hepcidin, IL-6 and preeclampsia in sub-Saharan Africa. The current study was conducted to evaluate iron status, hepcidin and IL-6 levels in Sudanese women with preeclampsia.

## Materials and methods

A case-control study was conducted at Saad Abuelela Maternity Hospital in Khartoum, Sudan, from June to December 2020. The cases were 60 pregnant women who presented with preeclampsia and have no history of pre-existing hypertension. Preeclampsia was defined as per the American College of Obstetricians and Gynaecologists criteria (ACOG Committee on Practice Bulletins—Obstetrics, 2020): pregnant women with onset of new hypertension (an average blood pressure reading of ≥140/90 mmHg taken on two occasions at least six hours apart) with proteinuria (≥ 300 mg/24 h) or features of end organ dysfunction in a previously normotensive woman. Preeclampsia was classified as severe in women with an average blood pressure reading of  ≥ 160/110 mmHg on two occasions or HELLP syndrome, which includes haemolysis, elevated liver enzymes and low platelet count; otherwise, preeclampsia was considered mild (ACOG Committee on Practice Bulletins—Obstetrics, 2020). The condition was also categorised as early presentation or late-onset preeclampsia, before and after 34  weeks, respectively (Tranquilli et al., 2013). Sixty healthy pregnant women without any systemic disease, such as hypertension, diabetes mellitus, renal disease or thyroid disease, served as a control for each preeclampsia case. Women with multiple pregnancies, diabetic women, smokers and women with fetuses who had major anomalies or died were excluded from both groups in the study.

After signing informed consent, the women were asked about their sociodemographic, obstetrics and clinical data, including age, parity, educational level, residence of antenatal attendance and history of miscarriage and preeclampsia/hypertension. Body mass index (BMI) was computed from the measured weight and height.

Then, 5 mL of blood was collected from each subject at the diagnosis and separated into two equal aliquots for blood and serum analysis. Haemoglobin levels were measured using a modern haematology analyser (Sysmex KX21n, Japan) according to the manufacturer’s instructions. The blood was then centrifuged and stored at −20°C until the assay of these elements. Serum ferritin was determined using the ferritin chemiluminescent immunoassay sandwich method [TOSOH instrument (AIA360), Japan]. Serum iron and total iron-binding capacity (TIBC) were measured using a colorimetric assay (Roche Diagnostics, Germany Cobas 311). Serum hepcidin and IL-6 concentrations were measured using an enzyme-linked immunosorbent assay according to the manufacturer’s instructions (Euroimmun, Lubeck, Germany).

The sample included 60 women in each group (ratio of 1:1) and was calculated using mean difference of 5 in the iron levels between the women who had preeclampsia and the healthy controls as reported before (Duvan et al., 2015). The sample size was used to achieve 80% power and a precision of 5%. It was assumed that 10% of the women would not respond or would provide incomplete data.

## Statistical analysis

The collected data were entered into SPSS version 22.0 for Windows for analysis. Continuous data were checked for normality using the Shapiro–Wilk test. The clinical data for the two groups (preeclampsia and controls) were compared using the Mann–Whitney *U* test for non-normally distributed data and Pearson’s chi-square (χ^2^) test for continuous and categorical data. Spearman correlations were performed between the continuous variables, and *p*-values of < 0.05 were considered significant.

## Results

During the study period, 60 pregnant women were enrolled in the case (preeclampsia) and 60 control groups. Nine women (15.0%) and four women (6.6%) had severe and early presented preeclampsia, respectively. There was no significant difference in the median (IQR) of age, parity or BMI between the two groups. However, the median (IQR) of gestational age was significantly lower in women with preeclampsia (Table 1).

**TABLE 1:** Comparing frequency (proportions) or median (interquartile range) of the sociodemographic and clinical variables between women with preeclampsia and controls in Khartoum Sudan, 2020.

Variables	Preeclampsia (60 women)	Controls (60 women)	p-value
Age, years	27 (24–31)	27 (25–32)	0.289
Parity	2 (1–4)	2 (1–3.75)	0.430
Body mass index, kg/m2	25.3 (22.9–26.4)	24.9 (22.0–25.7)	0.678
Gestational age, week	38.0 (37.0–39.0)	39.0 (38.0–40.0)	< 0.001
Residence			0.683
Urban	15 (25.0)	18 (30.0)	
Rural	45 (75.0)	42 (70.0)	
Education level			0.444
≤ secondary	49 (81.7)	53 (88.3)	
> secondary	11 (18.3)	7 (11.7)	
History of miscarriage			0.063
Yes	30 (50.0)	41 (68.3)	
No	30 (50.0)	19 (31.7)	
Antenatal care			0.706
≤ Two visits	36 (60.0)	39 (65.0)	
> Two visits	24 (40.0)	21 (35.0)	

There was no significant difference in residence, education, antenatal care, history of miscarriage or blood group. The median (IQR) of the iron status, hepcidin and interleukin-6, did not differ between the women with preeclampsia and the healthy controls (Table 2).

**TABLE 2:** Comparing the median (interquartile range) of the iron status indices, hepcidin, and interleukin-6 between women with preeclampsia and controls in Khartoum Sudan, 2020.

Variables	Preeclampsia (60 women)	Controls (60 women)	p-value
Hemoglobin, g/dl	11.9 (10.8–12.6)	11.6 (10.8–12.4)	0.665
Serum iron, mcg/dl	33.0 (24.0–53.0)	33.5 (26.2–51.0)	0.787
Serum ferritin, ng/mL	34.7 (21.2–51.2)	29.3 (19.3–39.6)	0.123
Total iron-binding capacity, mcg/dl	843.0 (720.0–854.0)	844.0 (831.0–860.0)	0.627
Unsaturated iron-biding capacity, mcg/dl	814.5 (699.0–819.0)	809.0 (798.0–817.0)	0.171
Hepcidin, ng/mL	54.0 (33.0–76.4)	48.0 (24.0–60.0)	0.195
Interleukin-6, pg/mL	8.1 (5.1–14.2)	6.3 (3.0–26.3)	0.445

There was no significant correlation between haemoglobin, hepcidin and IL-6, nor was there a significant correlation between serum iron, serum ferritin, hepcidin and IL-6. However, there was a significant positive correlation between hepcidin and IL-6 (r = 0.393, *p* = 0.002) (Table 3).

**TABLE 3:** Spearman correlation between the iron status, hepcidin, and interleukin-6 in pregnant women with preeclampsia in Khartoum Sudan, 2020.

Variables	Serum iron, mcg/dl		Serum ferritin, ng/mL		Hepcidin, ng/mL		Interleukin-6, pg/mL	
	*r*	*P*	*r*	*P*	*r*	*P*	*r*	*P*
Hemoglobin, g/dl	0.091	0.487	0.165	0.208	0.061	0.644	0.061	0.644
Serum iron, mcg/dl			0.222	0.208	0.133	0.312	−0.219	0.092
Serum ferritin, ng/mL					−0.020	0.877	0.103	0.432
Hepcidin, ng/mL							0.393	0.002

## Discussion

The main finding of the current study was that there was no difference in the levels of iron status, hepcidin and interleukin-6 between women with preeclampsia and healthy controls. This is in line with previous studies that showed no significant differences in maternal serum hepcidin levels in preeclampsia compared with age-matched controls (Cardaropoli et al., 2018). Duvan et al. (2015) reported no significant differences in pro-hepcidin, haemoglobin concentration, iron, ferritin or IL-6 in women with preeclampsia compared with controls.

On the other hand, several studies have shown higher serum iron and ferritin and serum hepcidin and pro-hepcidin levels in women with preeclampsia compared to healthy pregnant women (Muhsin et al., 2016; Brunacci et al., 2018; Shaji Geetha et al., 2022; Ölmez et al., 2022) It is worth mentioning that a recent meta-analysis (Bandyopadhyay et al., 2022), which included 760 individuals from seven studies, concluded that the pooled mean hepcidin levels were significantly higher in women with preeclampsia than in women without preeclampsia. Significantly lower iron-binding capacity, TIBC and transferrin levels have also been reported (Duvan et al., 2015).

Our results showed no significant difference in the levels of hepcidin and Il-6, and there was no significant correlation between the iron status, hepcidin and IL-6; however, we observed a significant positive correlation between hepcidin and IL-6. Some previous studies have reported higher levels of IL-6 in women with preeclampsia (Brunacci et al., 2018; Aggarwal et al., 2019). Our results (no significant correlation between iron status and hepcidin) were previously reported (Muhsin et al., 2016) among women with preeclampsia; however, in a later study, a positive correlation was found between iron status and hepcidin among women with normal pregnancies. Interestingly, Brunacci and colleagues reported significantly higher concentrations of serum iron, ferritin and transferrin saturation in women with preeclampsia; however, they reported lower hepcidin levels and no significant correlations between hepcidin concentration and iron status in these women (Brunacci et al., 2018).

It should be noted that our results should be compared to the results of other studies with caution. First, anaemia, which is highly prevalent (50%) among pregnant women, could have effects on the whole picture and the results of the iron status and hepcidin (Adam et al., 2018). Second, as previously mentioned, several communicable diseases (e.g., malaria and viral diseases) have been associated with preeclampsia in Sudan and these diseases could have influenced the iron status, hepcidin and IL6 (Adam et al., 2011; Ahmed et al., 2018, 2020). During states of acute or chronic inflammation, levels of hepcidin and other acute-phase reactants including ferritin increase in response to elevated IL-6 levels, leading to a decrease in serum iron levels as hepcidin levels rise. Increased hepcidin correlates with the pathophysiology of anaemia in chronic disease; the increase in inflammation causes a reduction in serum iron levels because the increase in hepcidin reduces iron transport out of cells (Chambers et al., 2021). In this study, ferritin levels were not correlated with IL-6, neither with hepcidin.

It has been reported that hepcidin is lower during pregnancy to ensure optimum iron bioavailability to the mother and fetus. However, inflammatory states, such as preeclampsia and parasitic (malaria) infection, have been associated with higher hepcidin levels during pregnancy (Koenig et al., 2014). The elevated hepcidin level in women with preeclampsia could be a protective mechanism to counteract iron overload (mediated cytotoxicity), oxidative stress and endothelial dysfunction that might occur in women with preeclampsia (Shaji Geetha et al., 2022).

For better interpretation of our findings, there are some limitations to be addressed. First; Sudan is endemic for many communicable diseases which is known to be associated with preeclampsia. However, we recruited apparently healthy pregnant women in both groups and did not screen for possible endemic diseases. Second; information regarding anaemia during entire course of pregnancy or before pregnancy is not available.

## Conclusion

In this study, women with preeclampsia had levels of iron homeostasis parameters, hepcidin and IL-6 similar to those observed in healthy pregnant women. There was no significant correlation between iron homeostasis parameters, hepcidin and IL-6.

## Data Availability

The raw data supporting the conclusion of this article will be made available by the authors, without undue reservation.
