# Corrigendum: Maternal serum iron status, hepcidin and interleukin-6 levels in women with preeclampsia

**DOI:** 10.3389/fphys.2024.1383996

**Published:** 2024-02-21

**Authors:** Yasir I. B. Ahmed, Hind S. Yagoub, Mohamed A. Hassan, I. Adam, Hamdan Z. Hamdan

**Affiliations:** ^1^ Faculty of Medical Laboratory Sciences, Omdurman Islamic University, Omdurman, Sudan; ^2^ Faculty of Medical Laboratory Sciences, College of Applied Medical Sciences, Taibah University, Madinah, Saudi Arabia; ^3^ Africa City of Technology, Khartoum, Sudan; ^4^ Department of Obstetrics and Gynaecology, Unaizah College of Medicine and Medical Sciences, Qassim University, Unaizah, Saudi Arabia; ^5^ Department of Basic Medical Sciences, Unaizah College of Medicine and Medical Sciences, Qassim University, Unaizah, Saudi Arabia; ^6^ Faculty of Medicine, Al-Neelain University, Khartoum, Sudan

**Keywords:** preeclampsia, hepcidin, iron homeostasis, interleukin-6, sub-Sahara Africa, pregnancy

In the published article, there was an error regarding the **Keywords**. Instead of “IL-6,” it should be “interleukin-6,” also kindly add the keyword “pregnancy” to the keyword list.

The authors apologize for this error and state that this does not change the scientific conclusions of the article in any way. The original article has been updated.

